# ﻿New species and records of *Cytospora* (Cytosporaceae, Diaporthales) from tree branches in Hebei Province, China

**DOI:** 10.3897/mycokeys.126.175474

**Published:** 2025-12-19

**Authors:** Tingqian Pei, Dianguang Xiong, Yingmei Liang

**Affiliations:** 1 The Key Laboratory for Silviculture and Conservation of the Ministry of Education, Beijing Forestry University, Beijing 100083, China Beijing Forestry University Beijing China; 2 Museum of Beijing Forestry University, Beijing Forestry University, Beijing 100083, China Beijing Forestry University Beijing China

**Keywords:** Canker disease, Cytosporaceae, multi-gene phylogeny, new species, new records

## Abstract

Species of *Cytospora* have been commonly reported as plant pathogens with wide host ranges and geographic distributions. In this study, ten strains of this genus were isolated from branches collected in Hebei Province, China. They were identified based on a multi-locus phylogeny of ITS, *act*, *rpb2*, *tef1-α*, and *tub2* genes, along with morphological characters. As a result, they were identified as six species, including five known species (*C. ampla*, *C. pseudochrysosperma*, *C. sophoricola*, *C. sorbariae*, and *C. yinchuanensis*) and one new species (*C. hebeiensis*). Among the known species, *C. ampla*, *C. sorbariae*, and *C. yinchuanensis* were newly discovered on *Malus spectabilis*; *C. pseudochrysosperma* was newly discovered on *Salix matsudana*; and *C. sophoricola* was newly discovered on *Caragana microphylla*. The results enrich the diversity of *Cytospora* species associated with tree canker and dieback diseases in Hebei Province, China.

## ﻿Introduction

*Cytospora* (Cytosporaceae, Diaporthales) was established by Ehrenberg with four species: *C. betulina*, *C. epimyces*, *C. resinae*, and *C. ribis* (Ehrenberg 1818). Subsequently, *C. chrysosperma*, which had been reported on *Populus nigra*, was designated as the type species (Donk 1964). Species of *Cytospora* can cause canker diseases in many woody plants, which can lead to weakness in growth or death of host plants (Sinclair et al. 1987; Adams et al. 2005; Fan et al. 2020; Stewart et al. 2022; Ilyukhin et al. 2023; Lin et al. 2023a, b).

*Cytospora* and its related sexual morphs, *Leucostoma*, *Valsa*, *Valsella*, and *Valseutypella*, were initially listed by early fungal literature for their identification (Fries 1823; Saccardo 1884; Kobayashi 1970; Barr 1978; Sutton 1980; Gvritishvili 1982; Spielman 1983, 1985). It was not until 2005 that Adams et al. proposed that *Valsa* was the only sexual type genus of *Cytospora*, and the other genera were treated as synonyms of *Valsa* (Adams et al. 2005). With the end of dual nomenclature for pleomorphic fungi (Wingfield et al. 2012; Crous et al. 2015), the single-name system, which employs the earliest published or most commonly used name, led to the retention of the older genus *Cytospora* over its sexual morph, *Valsa* (1849), on the list of protected fungi (McNeill et al. 2012; Fan et al. 2015a, b; Rossman et al. 2015).

Previously, morphological characteristics were employed to classify the sexual morph of *Cytospora*. Saccardo (1884) classified the genus *Valsa* into Macrosporae and Microsporae based on the size of ascospores. However, researchers suggested that stromata features are highly variable and have failed to provide reliable criteria to distinguish species of *Cytospora* (Urban 1958; Spielman 1985). Therefore, a method combining phylogenetics and morphology was introduced for related species identification. For instance, Adams et al. (2005, 2006) identified 62 *Cytospora* species from various hosts (e.g., *Eucalyptus*, *Malus*, *Pinus*) in South Africa using morphological and ITS-rDNA phylogenetic analyses, providing an identification key. Currently, researchers use morphology combined with multi-gene phylogenetic analyses to define species of *Cytospora*, and this approach has been progressively refined. Following the initial discovery of 14 new species by Norphanphoun et al. (2017) using four loci (ITS, LSU, *rpb2*, and *act*), Fan et al. (2020) enhanced phylogenetic resolution with the addition of *tef1-α* and *tub2*, clarifying *Cytospora* diversity and proposing thirteen new species and a new combination. The methodology was further validated by Jia et al. (2023) in a limited geographical area (Fengtai, Beijing). Subsequently, Lin et al. (2024) established a taxonomic framework based on five genetic loci (ITS, *act*, *rpb2*, *tef1-α*, *tub2*) and constructed a new morphological grouping system: three asexual morphological groups (including thirteen types, a1–a13) and three sexual morphological groups (including eight types, s1–s8). Recently, Jiang et al. (2025a) classified *Cytospora* into ten species complexes and twelve singletons, providing valuable information for future research.

This study delineates the taxonomic status of ten *Cytospora* isolates from diseased branches in Hebei Province, China, with comprehensive descriptions, microscopic photographs, and updated phylogenetic trees, along with one new species and five new host records.

## ﻿Materials and methods

### ﻿Sample collection and isolation

**Figure 1.**
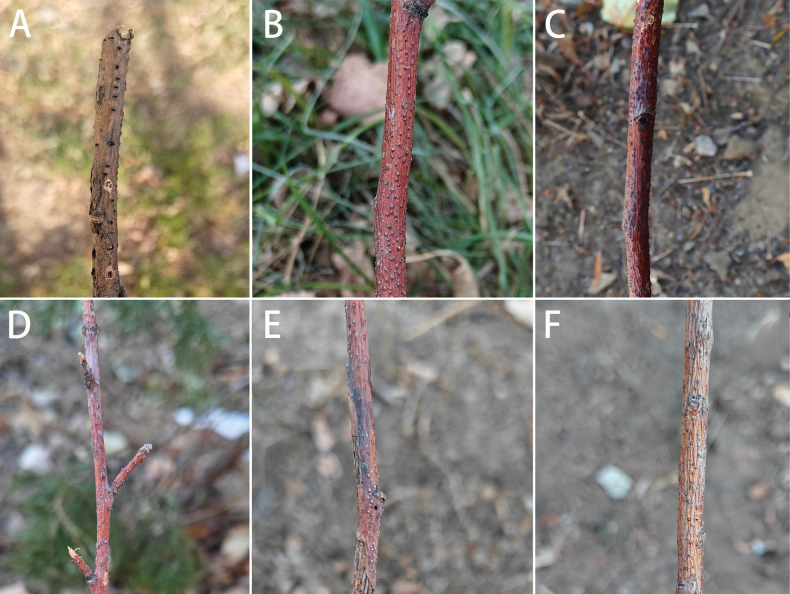
Disease symptoms from different tree branches. **A.***Caragana microphylla*; **B, C.***Malus pumila*; **D, E.***M. spectabilis***F.***Salix matsudana*.

Ten specimens were collected from diseased branches of woody hosts distributed in Hebei Province. Sampled trees expressed general symptoms and signs of canker diseases, including elongated, slightly sunken, and discolored areas in the bark; several prominent, dark conidiomata and ascomata immersed in the bark; and erumpent fruiting bodies breaking through the bark surface when mature. The bark appeared yellow, brown, reddish brown, gray, or black, becoming watery or odorous as the tissues deteriorated (Fig. 1).

A total of ten isolates were obtained by removing mucoid spore mass from conidiomata and ascomata, spreading the suspension on the surface of potato dextrose agar (potato, 200 g; glucose, 20 g; agar, 20 g; distilled water to complete 1000 mL) in a Petri dish, and incubating at 25 °C in the dark. After discrete colonies grew on the plate, a small piece of PDA block was cut from the edge of a well-isolated single colony and transferred to the center of a fresh PDA plate to obtain a pure culture. All specimens are deposited in the Museum of the Beijing Forestry University (**BJFC**), and the cultures are maintained in the China Forestry Culture Collection Center (**CFCC**; https://cfcc.caf.ac.cn/).

### ﻿Morphological observation

The study of *Cytospora* involved morphological observations of fruiting bodies growing on tree bark surfaces. Macromorphological features were photographed using a Leica stereomicroscope (M205 FA) (Leica Microsystems, Wetzlar, Germany), including the arrangement and size of stromata; the presence or absence of a conceptacle; the size, color, and shape of discs; and the diameter of ostioles. Micromorphological features were photographed using a Nikon Eclipse 80i microscope (Nikon Corporation, Tokyo, Japan), including the size and shape of conidiophores, asci, and conidia/ascospores. We measured at least ten conidiostromata/ascostromata, 30 asci, and 50 conidia/ascospores to calculate the mean size. Colony diameters were measured, and the colony colors were described according to the color charts of Rayner (1970).

### ﻿DNA extraction, PCR amplification, and sequencing

Colonies used for DNA extraction were grown on PDA for five days and obtained from the surface by scraping. The genomic DNA was extracted using the cetyltrimethylammonium bromide (CTAB) method (Doyle and Doyle 1990). DNA products were stored at −20 °C. The PCR mixture volume was 20 µL, which consisted of 10 µL Mix (Promega), 7 µL double deionized water, 1 µL each pre- and post-primer, and 1 µL DNA template. Five loci (ITS, *act*, *rpb2*, *tef1-α*, and *tub2*) were used for comparison-based phylogenetic analyses to determine the identities of the isolates. The primers and PCR conditions used in the current study are listed in Table 1. Amplified PCR products were sent to a commercial sequencing provider (Tsingke Biotechnology Co. Ltd., Beijing, China). The forward and reverse sequences were edited and assembled using SeqMan v.7.1.0 software. The sequences obtained in this study were deposited in GenBank (http://www.ncbi.nlm.nih.gov).

**Table 1.** Genes used in this study with PCR primers.

Locus	PCR primers	Thermal cycles	References
ITS	ITS1/ITS4	(95 °C: 30 s, 51 °C: 30 s, 72 °C: 1min) × 35 cycles	White et al. (1990)
*act*	ACT-512F/ACT-783R	(95 °C: 15 s, 55 °C: 20 s, 72 °C: 1min) × 35 cycles	Carbone and Kohn (1999)
*rpb2*	RPB2-5F/RPB2-7cR	(95 °C: 30 s, 55 °C: 30 s, 72 °C: 1min) × 35 cycles	Liu et al. (1999)
*tef1-α*	EF1-728F/EF1-1567R	(95 °C: 45 s, 55 °C: 45 s, 72 °C: 1min) × 35 cycles	Rehner et al. (2005)
*tub2*	Bt2a/Bt2b	(95 °C: 30 s, 52 °C: 60 s, 72 °C:1 min) × 35 cycles	Glass and Donaldson (1995)

### ﻿Phylogenetic tree

The phylogenetic analysis was performed based on a combined dataset of sequences to compare *Cytospora* species from the current study with other sequences obtained from GenBank (Table 2). The sequence datasets used in this study were based on Jiang et al. (2025a), and outgroup taxa were set as follows: *C. brunnea* (CFCC 71082) and *C. viticola* (CBS 141605) for the *Chrysosperma* SC, and *C. donglingensis* (CFCC 53159) and *C. viticola* (CBS 141605) for the Ribis SC.

**Table 2.** Strains of *Cytospora* used in the molecular analyses in *Chrysosperma* and *Ribis* SCs. NA: not applicable. Strains in this study are marked in bold, and * indicates ex-type strains.

Species	Strain numbers	Host	GenBank accession numbers					References
			ITS	*act*	*rpb2*	*tef1-α*	*tub2*	
*Cytospora ailanthicola*	CFCC 89970*	*Ailanthus altissima*	MH933618	MH933526	MH933592	MH933494	MH933565	Jiang et al. (2025a)
*C. ailanthicola*	CFCC 58712	*Salix matsudana*	PQ778497	PV454710	PV461889	PV467118	PV467242	Jiang et al. (2025a)
*C. ailanthicola*	CFCC 58713	*Salix chaenomeloides*	PQ778498	PV454711	PV461890	PV467119	PV467243	Jiang et al. (2025a)
*C. ailanthicola*	CFCC 71105	*Salix* sp.	PQ778508	PV454719	PV461900	PV467129	PV467253	Jiang et al. (2025a)
*C. ampla*	CFCC 71189*	*Rubus biflorus*	PQ778511	PV454722	PV461903	PV467132	PV467256	Jiang et al. (2025a)
*C. ampla*	CFCC 71044*	*Salix* sp.	PQ778509	PV454720	PV461901	PV467130	PV467254	Jiang et al. (2025a)
***C. ample***	**CFCC 72627**	***Salix matsudana***	**PV715411**	**PV702606**	**PV714133**	**PV766892**	**PV745444**	**This study**
***C. ample***	CFCC 72603*	***Malus spectabilis***	**PV715414**	**PV702609**	**NA**	**PV766894**	**NA**	**This study**
***C. ample***	**CFCC 72604**	***Malus spectabilis***	**PV715416**	**NA**	**NA**	**PV766896**	**NA**	**This study**
*C. annulata*	CBS 118089	*Acer rubrum*	PP988738	PQ074598	PQ074911	PQ074273	PQ075228	Lin et al. (2024)
*C. auerswaldii*	CBS 153.29	*unknown*	PP988740	PQ074600	PQ074913	PQ074275	PQ075230	Lin et al. (2024)
*C. betulae*	CBS 141622*	*Betula papyrifera*	PP988752	PQ074610	PQ074922	PQ074284	PQ075236	Lin et al. (2024)
*C. brunnea*	CFCC 71082*	*Prunus mira*	PQ778532	PV454738	PV461923	PV467153	PV467277	Jiang et al. (2025a)
*C. carbonacea*	CFCC 89947	*Ulmus pumila*	KR045622	KP310842	KU710950	KP310855	KP310825	Fan et al. (2020)
*C. chrysosperma*	CBS 120	*Populus balsamifera*	PP988773	PQ074626	PQ074942	PQ074304	PQ075255	Lin et al. (2024)
*C. chrysosperma*	CBS 197.50*	*Populus tremula*	PP988777	PQ074630	PQ074946	PQ074308	PQ075259	Lin et al. (2024)
*C. chungenii*	CFCC 71027*	*Hippophae rhamnoides*	PQ778534	PV454740	PV461925	PV467155	PV467279	Jiang et al. (2025a)
*C. chungenii*	CFCC 71310*	*Hippophae rhamnoides*	PQ778535	PV454741	PV461926	PV467156	PV467280	Jiang et al. (2025a)
*C. coryli*	CFCC 53162*	*Corylus mandshurica*	MN854450	NA	MN850751	MN850758	MN861120	Zhu et al. (2020)
*C. crataegina*	CFCC 56027*	*Crataegus pinnatifida*	PP988791	PQ074642	PQ074958	PQ074321	PQ075271	Lin et al. (2024)
*C. crataegina*	CFCC 56029	*Crataegus pinnatifida*	PP988793	PQ074644	PQ074960	PQ074323	PQ075273	Lin et al. (2024)
*C. diminuta*	CFCC 71034*	*Sophora moorcroftiana*	PQ778540	PV454746	PV461931	PV467161	PV467285	Jiang et al. (2025a)
*C. diminuta*	CFCC 71312*	*Sophora moorcroftiana*	PQ778541	PV454747	PV461932	PV467162	PV467286	Jiang et al. (2025a)
*C. donglingensis*	CFCC 53159*	*Platycladus orientalis*	MW418412	MW422903	MW422915	MW422927	MW422939	Pan et al. (2018)
*C. eastringensis*	CFCC 58222*	*Populus adenopoda*	PP988818	NA	PQ074980	PQ074346	NA	Lin et al. (2024)
*C. elaeagni*	CFCC 58241	*Elaeagnus angustifolia*	PP988819	PQ074661	PQ074981	PQ074347	NA	Lin et al. (2024)
*C. euonymicola*	CFCC 50499*	*Euonymus kiautschovicus*	MH933628	MH933535	MH933598	MH933503	MH933570	Fan et al. (2020)
*C. fengtaiensis*	CFCC 59449*	*Acer palmatum*	OR826167	OR832000	OR832022	OR832044	OR832064	Jia et al. (2023)
*C. fugax*	CBS 203.42*	*Salix* sp.	PP988848	PQ074686	PQ075006	NA	PQ075318	Adams et al. (2006)
*C. fugax*	CFCC 71307	*Salix takasagoalpina*	PQ778546	PV454752	PV461937	PV467167	PV467291	Jiang et al. (2025a)
*C. gigaloculata*	CFCC 89620*	*Juglans regia*	KR045628	KU710997	KU710957	KU710920	KR045669	Fan et al. (2020)
*C. gigaloculata*	CFCC 71097	*Rosa multiflor*	PQ778547	PV454753	PV461938	PV467168	PV467292	Jiang et al. (2025a)
*C. gigaloculata*	CFCC 71098	*Rosa multiflor*	PQ778548	PV454754	PV461939	PV467169	PV467293	Jiang et al. (2025a)
*C. globosa*	CBS 118976	*Abies alba*	PP988851	PQ074689	PQ075009	PQ074376	PQ075321	Lin et al. (2024)
*C. globosa*	MFLUCC 16-1153*	*Abies alba*	MT177935	NA	MT432212	MT454016	NA	Li et al. (2020)
*C. guyuanensis*	CFCC 55855*	*Salix* sp.	PP988853	NA	PQ075011	PQ074378	PQ075323	Lin et al. (2024)
*C. haidianensis*	CFCC 54057*	*Euonymus alatus*	MT360042	MT363979	MT363988	MT363998	MT364008	Zhou et al. (2020)
***C. hebeiensis***	CFCC 72601*	***Malus pumila***	**PV715410**	**PV702605**	**PV714132**	**PV766891**	**PV745443**	**This study**
*C. hejingensis*	CFCC 59571*	*Salix* sp.	PP060455	PP059657	PP059663	PP059667	PP059673	Wang et al. (2024)
*C. hippophaes*	CBS 259.88	*Hippophae rhamnoides*	PP988856	NA	PQ075014	PQ074381	PQ075326	Lin et al. (2024)
*C. hippophaes*	CFCC 58943	*Hippophae rhamnoides*	PP988858	PQ074692	PQ075016	PQ074383	PQ075327	Lin et al. (2024)
*C. hippophaes*	CFCC 71064	*Hippophae rhamnoides*	PQ778552	NA	PV461943	PV467172	PV467297	Jiang et al. (2025a)
*C. iranica*	IRAN 4200C*	*Malus domestica*	MW295652	MZ014512	MW824359	MW394146	NA	Hanifeh et al. (2022)
*C. joaquinensis*	CBS 144235	*Populus deltoides*	MG971895	MG972044	NA	MG971605	NA	Lawrence et al. (2018)
*C. juniperina*	CFCC 50501*	*Juniperus przewalskii*	MH933632	MH933539	MH933602	MH933507	NA	Fan et al. (2020)
*C. leucosperma*	CBS 109491	*Fagus sylvatica*	PP988884	PQ074714	PQ075035	PQ074407	PQ075347	Lin et al. (2024)
*C. lhasaensis*	CFCC 59094*	*Rosa omeiensis*	OR769863	OR767319	OR767333	OR767359	OR767347	Li et al. (2024)
*C. lhasaensis*	CFCC 71113	*Rosa sericea*	PQ778558	PV454762	PV461949	PV467178	PV467301	Jiang et al. (2025a)
*C. linzhiensis*	CFCC 71045*	*Euonymus japonicus*	PQ778562	PV454765	PV461952	PV467181	PV467304	Jiang et al. (2025a)
*C. linzhiensis*	CFCC 71179*	*Alnus nepalensis*	PQ778563	PV454766	PV461953	PV467182	PV467305	Jiang et al. (2025a)
*C. longispora*	CBS 144236*	*Prunus domestica*	MG971905	MG972054	NA	MG971615	NA	Fan et al. (2020)
*C. longistiolata*	MFLUCC 16-0628	Salix × fragilis	KY417734	KY417700	KY417802	NA	NA	Norphanphoun et al. (2017)
*C. macropycnidia*	CBS 149338*	*Vitis vinifera*	OP038094	OP003977	OP095265	OP106954	OP079909	Travadon et al. (2022)
*C. malvicolor*	CFCC 56567*	*Corylus mandshurica*	PP988915	PQ074744	PQ075063	PQ074438	PQ075377	Lin et al. (2024)
*C. malvicolor*	CFCC 56577*	*Corylus mandshurica*	PP988916	PQ074745	PQ075064	PQ074439	PQ075378	Lin et al. (2024)
*C. melnikii*	CFCC 89984	*Rhus typhina*	MH933644	MH933551	MH933609	MH933515	MH933580	Fan et al. (2020)
*C. mougeotii*	CBS 198.50	*Picea abies*	PP988918	PQ074747	PQ075066	PQ074441	PQ075380	Lin et al. (2024)
*C. neolhasaensis*	CFCC 58706*	*Salix wallichiana*	PP988902	PQ074732	PQ075052	PQ074425	PQ075365	Jiang et al. (2025a)
*C. neolhasaensis*	CFCC 58862*	*Salix wallichiana*	PQ778576	PV454777	PV461963	PV467192	PV467315	Jiang et al. (2025a)
*C. nobilis*	CFCC 58227	*Laurus nobilis*	PP988928	PQ074756	PQ075075	PQ074449	PQ075389	Lin et al. (2024)
*C. nobilis*	CFCC 58228	*Laurus nobilis*	PP988929	PQ074757	PQ075076	PQ074450	PQ075390	Lin et al. (2024)
*C. nobilis*	CFCC 71102	*Salix takasagoalpina*	PQ778577	PV454778	PV461964	PV467193	PV467316	Jiang et al. (2025a)
*C. nobilis*	CFCC 71414	*Salix takasagoalpina*	PQ778578	PV454779	PV461965	PV467194	PV467317	Jiang et al. (2025a)
*C. piceae*	CFCC 52841*	*Picea crassifolia*	MH820398	MH820406	MH820395	MH820402	MH820387	Pan et al. (2018)
*C. pinastri*	CBS 113.81	*Abies alba*	PP988941	PQ074768	PQ075087	PQ074461	PQ075401	Lin et al. (2024)
*C. platycladicola*	CFCC 50038*	*Platycladus orientalis*	KT222840	MH933555	MH933613	MH933519	MH933584	Fan et al. (2020)
*C. populina*	CFCC 89644*	*Salix psammophila*	KF765686	KU711007	KU710969	KU710930	KR045681	Fan et al. (2020)
*C. populinopsis*	CFCC 50032*	*Sorbus aucuparia*	MH933648	MH933556	MH933614	MH933520	MH933585	Fan et al. (2020)
*C. prunicola*	MFLU 17-0995*	*Prunus* sp.	MG742350	MG742353	MG742352	NA	NA	Norphanphoun et al. (2017)
*C. pseudochrysosperma*	CFCC 54081*	*Populus* sp.	MZ702631	NA	NA	OK303613	OK303680	Lin et al. (2024)
*C. pseudochrysosperma*	CFCC 89981*	Populus alba var. pyramidalis	MH933625	MH933533	MH933597	MH933501	MH933568	Lin et al. (2024)
***C. pseudochrysosperma***	**CFCC 72617**	***Salix babylonica***	**PV715412**	**PV702607**	**PV714134**	**PV766893**	**PV745445**	**This study**
***C. pseudochrysosperma***	**CFCC 72613**	***Salix babylonica***	**PV715413**	**PV702608**	**PV714135**	**NA**	**PV745446**	**This study**
*C. qinghaiensis*	CFCC 50026*	*Ulmus pumila*	KP281267	KP310843	KU710972	KP310856	KP310826	Lin et al. (2024)
*C. qingshuiensis*	CFCC 56268*	*Platycladus orientalis*	PP988956	PQ074782	PQ075099	NA	NA	Lin et al. (2024)
*C. qingshuiensis*	CFCC 56349*	*Platycladus orientalis*	PP988957	PQ074783	PQ075100	PQ074474	PQ075415	Lin et al. (2024)
*C. qingshuiensis*	ZHKUCC 23-0978	*Malus domestica*	PP829299	PP850109	PP839460	NA	PP839451	Lin et al. (2024)
*C. ribis*	CBS 187.36	*Ribes rubrum*	PP988963	PQ074788	PQ075106	PQ074480	PQ075420	Adams et al. (2006)
*C. rosigena*	MFLUCC 18-0921	*Rosa* sp.	MN879872	NA	NA	NA	NA	Li et al. (2020)
*C. rostrata*	CFCC 89909*	*Salix cupularis*	KR045643	KU711009	KU710974	NA	NA	Lin et al. (2024)
*C. rostrata*	CFCC 89910	*Salix cupularis*	KR045644	KU711010	KU710975	KU710933	NA	Lin et al. (2024)
*C. saccardoi*	CBS 109752R	*Juniperus communis*	PP988975	NA	NA	PQ074492	PQ075430	Lin et al. (2024)
*C. salicacearum*	MFLUCC 15-0509*	*Salix alba*	KY417746	KY417712	KY417814	NA	NA	Fan et al. (2020)
*C. salicina*	CBS 507.77	*unknowm*	PP988981	PQ074804	PQ075122	PQ074497	PQ075435	Lin et al. (2024)
*C. salicina*	MFLUCC 15-0862*	*Salix alba*	KY417750	KY417716	KY417818	NA	NA	Fan et al. (2020)
*C. sanbaensis*	CFCC 58242*	*Populus adenopoda*	PP988983	PQ074806	PQ075124	PQ074499	PQ075437	Lin et al. (2024)
*C. schulzeri*	MFLUCC 15-0507*	*Malus domestica*	KY417740	KY417706	KY417808	NA	NA	Norphanphoun et al. (2017)
*C. shaanxiensis*	CFCC 56032*	*Lindera obtusiloba*	PP988987	PQ074810	PQ075128	PQ074502	PQ075441	Lin et al. (2024)
*C. sidaohensis*	CFCC 56042*	*Corylus heterophylla*	PP988992	PQ074815	PQ075133	PQ074507	PQ075446	Lin et al. (2024)
*C. sinensis*	CFCC 58231	*Populus simonii*	PP988995	PQ074818	PQ075136	PQ074510	PQ075449	Lin et al. (2024)
*C. sinensis*	CFCC 58235*	*Populus simonii*	PP988997	PQ074820	PQ075138	PQ074512	PQ075451	Lin et al. (2024)
*C. songshanensis*	CFCC 56351*	*Platycladus orientalis*	PP989006	PQ074828	PQ075147	PQ074521	PQ075459	Lin et al. (2024)
*C. sophorae*	CFCC 50048	*Magnolia grandiflora*	MH820401	MH820409	MH820397	MH820405	MH820390	Fan et al. (2020)
*C. sophorae*	CFCC 89598	*Styphnolobium japonicum*	KR045654	KU711018	KU710985	KU710941	KR045695	Fan et al. (2020)
*C. sophoricola*	CFCC 89595*	*Styphnolobium japonicum*	KR045655	KU711019	KU710986	KU710942	KR045696	Fan et al. (2020)
***C. sophoricola***	**CFCC 72610**	***Caragana microphylla***	**PV715409**	**PV702604**	**PV714131**	**PV766890**	**PV745442**	**This study**
*C. sophoriopsis*	CFCC 58464	*Populus szechuanica*	PP989009	PQ074831	PQ075150	PQ074524	PQ075461	Lin et al. (2024)
*C. sorbariae*	CFCC 56025*	*Corylus heterophylla*	PP988788	PQ074639	PQ074955	PQ074318	PQ075268	Jia et al. (2023)
*C. sorbariae*	CFCC 59445*	*Sorbaria sorbifolia*	OR826176	OR832009	OR832031	OR832053	OR832073	Jia et al. (2023)
***C. sorbariae***	**CFCC 72609**	***Malus spectabilis***	**PV715417**	**NA**	**NA**	**NA**	**PV745448**	**This study**
*C. suecica*	CBS 450.51*	*Populus tremula*	PP989015	PQ074834	PQ075156	PQ074530	PQ075467	Lin et al. (2024)
*C. tanaitica*	MFLUCC 14-1057*	*Betula pubescens*	KT459411	KT459413	NA	NA	NA	Ariyawansa et al. (2015)
*C. tetraspora*	CFCC 55847*	*Quercus aliena*	PP989021	PQ074840	PQ075162	PQ074536	PQ075473	Lin et al. (2024)
*C. tetraspora*	CFCC 56279*	*Tilia mongolica*	PP989023	PQ074842	PQ075164	PQ074538	PQ075475	Lin et al. (2024)
*C. tritici*	CBS 118561	*Populus simonii*	PP989038	PQ074856	PQ075175	PQ074549	PQ075486	Lin et al. (2024)
*C. tritici*	CBS 118563	*Populus nigra* cv. *italica*	PP989039	PQ074857	PQ075176	PQ074550	PQ075487	Lin et al. (2024)
*C. ulmi*	MFLUCC 15-0863*	*Xylocarpus moluccensis*	KY417759	KY417725	KY417827	NA	NA	Norphanphoun et al. (2017)
*C. ulmicola*	MFLUCC 18-1227*	*Xylocarpus moluccensis*	MH940220	MH940216	NA	NA	NA	Phookamsak et al. (2019)
*C. verrucosa*	CFCC 53157 *	*Platycladus orientalis*	MW418408	NA	MW422911	MW422923	MW422935	Pan et al. (2021)
*C. viticola*	CBS 141605	*Vitis vinifera*	PP989058	PQ074868	PQ075185	PQ074562	PQ075497	Travadon et al. (2022)
*C. washingtonensis*	CBS 141619*	*Crataegus* sp.	PP989065	PQ074874	PQ075192	PQ074569	PQ075502	Lin et al. (2024)
*C. xinjiangensis*	CFCC 53183*	*Rosa* sp.	MK673065	MK673035	MK673005	MK672952	MK672981	Pan et al. (2020)
*C. yakimana*	CBS 149297*	*Vitis vinifera*	OM976602	ON012555	ON045093	ON012569	ON086750	Travadon et al. (2022)
*C. yinchuanensis*	CFCC 50040*	*Malus domestica*	KR045649	KU711013	KU710980	KU710936	KR045690	Lin et al. (2024)
*C. yinchuanensis*	CFCC 50042*	*Malus asiatica*	KR045650	KU711014	KU710981	KU710937	KR045691	Lin et al. (2024)
***C. yinchuanensis***	**CFCC 72611**	***Malus spectabilis***	**PV715415**	**PV702610**	**NA**	**PV766895**	**PV745447**	**This study**
***C. yinchuanensis***	**CFCC 72612**	***Malus pumila***	**PV715418**	**NA**	**NA**	**NA**	**NA**	**This study**
*C. yingmeiae*	CFCC 71203*	*Quercus semecarpifolia*	PQ778629	PV454829	PV462013	PV467238	PV467361	Jiang et al. (2025a)
*C. yingmeiae*	CFCC 71308*	*Quercus semecarpifolia*	PQ778630	PV454830	NA	PV467239	NA	Jiang et al. (2025a)
*C. zhaitangensis*	CFCC 56227*	*Euonymus japonicus*	OQ344750	OQ410623	OQ398733	OQ398760	OQ398789	Lin et al. (2024)
*C. zhaitangensis*	CFCC 58608	*Corylus mandshurica*	PP989073	PQ074882	PQ075199	PQ074577	PQ075509	Lin et al. (2024)

All sequences were aligned in MAFFT v. 7 on the web server (https://mafft.cbrc.jp/alignment/server/) (Katoh and Standley 2013; Katoh et al. 2019) and adjusted in MEGA v. 7.0 (Tamura et al. 2013). Alignments excluded ambiguous regions. Multi-gene phylogenetic analyses employing Maximum Likelihood (ML) analysis (Guindon et al. 2010) and Bayesian inference (BI) analysis (Ronquist et al. 2012) were conducted using PhyML v. 3.0 and MrBayes v. 3.1.2 software, respectively. The phylogenetic tree was visualized with FigTree v. 1.4.0 (http://tree.bio.ed.ac.uk/software/figtree/) and additionally edited with Adobe Illustrator CS v. 5 (Adobe Systems Inc., USA). Maximum Likelihood bootstrap values (MLBP) ≥ 75% and Bayesian posterior probabilities (BPP) ≥ 0.90 are shown for each tree.

## ﻿Results

### ﻿Phylogenetic analyses

Phylogenetic analyses placed the ten isolates into two species complexes, two in the *Chrysosperma* SC and eight in the *Ribis* SC. To better resolve their relationships, separate phylogenetic trees were constructed for each complex.

**Figure 2.**
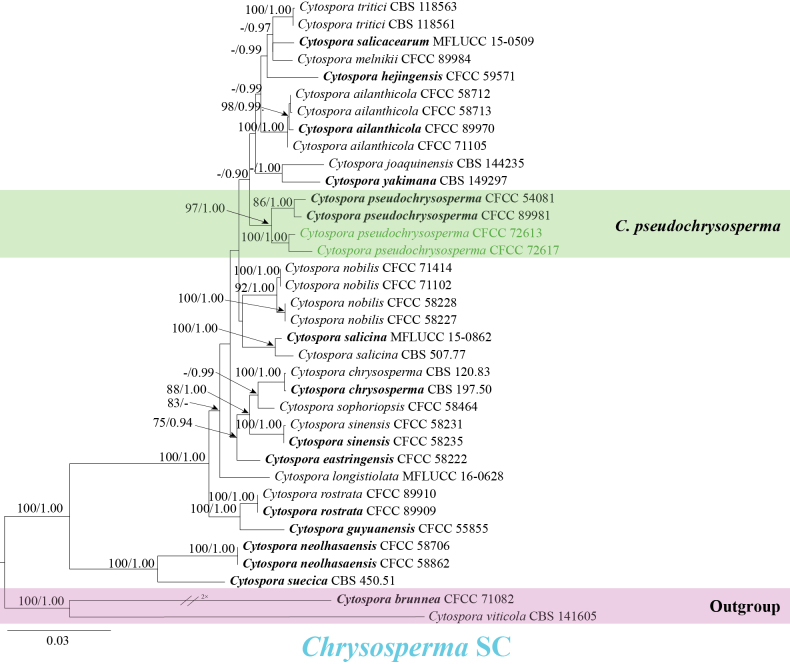
Phylogenetic tree inferred from ML analysis based on combined ITS, *act*, *rpb2*, *tef1-α*, and *tub2* sequence data of the *Chrysosperma* SC. The tree was rooted with *Cytospora brunnea* (CFCC 71082) and *C. viticola* (CBS 141605). Numbers above the branches indicate MLBP ≥ 75% and BPP ≥ 0.90. Ex-type isolates are marked in bold. Isolates in this study are highlighted in green.

In the *Chrysosperma* SC, the gene loci ITS, *act*, *rpb2*, *tef1-α*, and *tub2* were combined and analyzed to infer the phylogenetic placement of two isolates. The dataset consisted of 36 sequences, including the outgroup *Cytospora brunnea* (CFCC 71082) and *C. viticola* (CBS 141605). *Cytospora* ingroup strains had a total of 2,846 characters, including gaps (523 characters for ITS, 267 for *act*, 728 for *rpb2*, 590 for *tef1-α*, and 738 for *tub2*). MLBP ≥ 75% and BPP ≥ 0.90 are shown above the branches. For ML analysis, the substitution model (GTR+G+I model) for each dataset was selected following recent studies (Pan et al. 2018, 2020; Fan et al. 2020). Confidence levels for the nodes were determined using 1,000 replicates of bootstrapping methods (Hillis and Bull 1993). The matrix had 757 distinct alignment patterns. Estimated base frequencies were as follows: A = 0.250457, C = 0.289689, G = 0.236963, T = 0.222891; substitution rates: AC = 1.108390, AG = 3.318151, AT = 1.367714, CG = 0.680654, CT = 6.041623, GT = 1.000000; gamma distribution shape parameter: α = 0.250954. The two isolates aggregated with *C. pseudochrysosperma* (Fig. 2).

**Figure 3.**
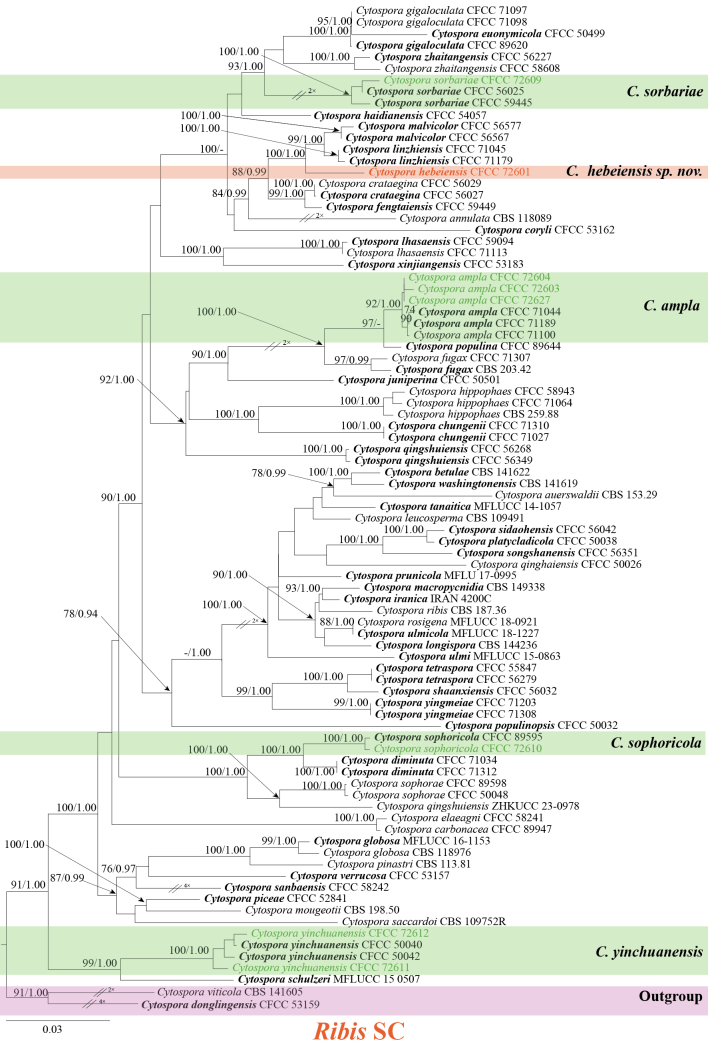
Phylogenetic tree inferred from ML analysis based on combined ITS, *act*, *rpb2*, *tef1-α*, and *tub2* sequence data of the *Ribis* SC. The tree was rooted with *Cytospora donglingensis* (CFCC 53159) and *C. viticola* (CBS 141605). Numbers above the branches indicate MLBP ≥ 75% and BPP ≥ 0.90. Ex-type isolates are in bold. Isolates in this study are highlighted in two different colors, with the new species shown in orange and the known species shown in green.

In the *Ribis* SC, the dataset of *Cytospora* isolates consisted of 87 sequences, using *Cytospora donglingensis* (CFCC 53159) and *C. viticola* (CBS 141605) as the outgroup. *Cytospora* ingroup strains had a total of 2,951 characters, including gaps (546 characters for ITS, 320 for *act*, 721 for *rpb2*, 619 for *tef1-α*, and 745 for *tub2*). The phylogenetic tree similarly shows MLBP ≥ 75% and BPP ≥ 0.90 above the branches. The ML analysis also employed the GTR+G+I model, and confidence levels for the nodes were determined using 1,000 replicates of bootstrapping methods (Hillis and Bull 1993). The matrix had 1,367 distinct alignment patterns. Estimated base frequencies were as follows: A = 0.245854, C = 0.287010, G = 0.236346, T = 0.230790; substitution rates: AC = 1.271593, AG = 3.563785, AT = 1.403228, CG = 0.929449, CT = 6.467342, GT = 1.000000; gamma distribution shape parameter: α = 0.261475. Seven isolates were identified as known species: *C. ampla*, *C. sophoricola*, *C. sorbariae*, and *C. yinchuanensis*. One isolate formed a separate clade, representing a new species, which we named *C. hebeiensis* (Fig. 3).

### ﻿Taxonomy

#### ﻿ Cytospora ampla

Taxon classificationFungiDiaporthalesValsaceae

Ning Jiang, Persoonia 55: 389 (2025)

7685B76B-6B5C-568D-8E8C-A3C975965CD1

Fig. 4

##### Description.

***Sexual morph*: *Stromata*** Group SIII (type s8), immersed in the bark, erumpent through the surface when mature, without extending to a large circular area. ***Conceptacle*** absent. ***Disc*** light grey, surrounded by ostiolar, circular to ovoid, 64–206 µm in diam, with 12–18 ostioles irregularly circularly in the disc. ***Ostioles*** umber to black when mature, arranged regularly in a disc, flask-shaped to spherical, 73–201 µm in diam. ***Asci*** hyaline, with a chitinoid, refractive ring, clavate to elongate-obovoid, 60–77 × 11–13 (av. = 46.4 ± 1.9 × 9.4 ± 0.7, n = 30) µm, 4-spored. ***Ascospores*** hyaline, elongate-allantoid, thin-walled, aseptate, 18.8–24.0 × 4.5–6.2 (av. = 20.2 ± 0.4 × 5.3 ± 0.8, n = 50) µm. ***Asexual morph***: not observed.

##### Culture characteristics.

Colonies initially white and entirely covering the 6 cm Petri dish after 5 d, becoming olivaceous buff to slight helical after 14 d. The colonies are flat, felt-like, thin with a uniform texture.

##### Materials examined.

China, Hebei Province, Saihanba, 42°23'33"N, 117°22'17"E, from branches of *Malus spectabilis*, 11 September 2024, C.M. Tian, T.Q. Pei & M.H. Wang (BJFC-S2549, living cultures CFCC 72603; BJFC-S2550, living culture CFCC 72604); 42°23'33"N, 117°22'17"E, from branches of *Salix matsudana*, 8 July 2024, C.M. Tian, T.Q. Pei & Y.Y. Wu (BJFC-S2551, living culture CFCC 72627).

**Figure 4.**
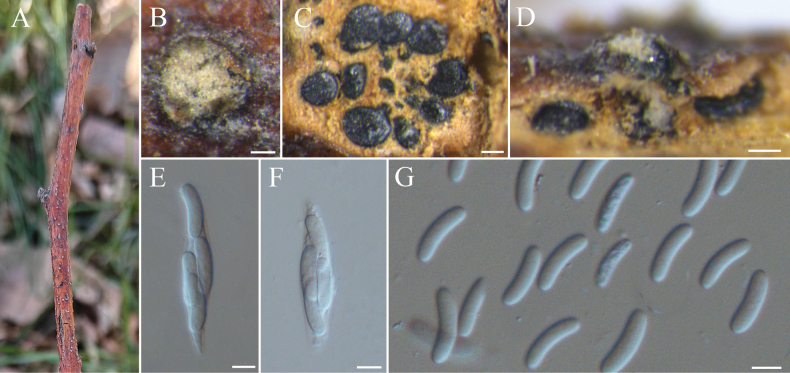
*Cytospora ampla* (BJFC-S2549). **A, B.** Habit of pseudostromata on branch; **C.** transverse section through pseudostroma with ascomata; **D.** Longitudinal section through pseudostroma with ascomata; **E, F.** Asci; **G.** Ascospores. Scale bars: 100 μm (**B–D**); 10 μm (**E–G**).

##### Notes.

*Cytospora ampla* was initially isolated from branches of *Salix* sp. and *Rubus biflorus* in Xizang, China. This species is characterized by its sexual morphs having four-spored asci (Jiang et al. 2025a). In the present study, we obtained three new isolates (CFCC 72603, CFCC 72604, and CFCC 72627) from *Malus spectabilis* and *Salix matsudana*. These isolates clustered robustly with the ex-type isolate CFCC 71044 (Fig. 3) with high support (MLBP/BPP = 92/1.00), confirming their identification as *C. ampla*. Therefore, this represents a new host record (*M. spectabilis*) for *C. ampla* and its first occurrence in Hebei Province, China.

#### ﻿ Cytospora hebeiensis

Taxon classificationFungiDiaporthalesValsaceae

T.Q. Pei & Y.M. Liang sp. nov.

57FC8F0A-FA4A-5980-BC4B-BE2C77E8F296

858825

Fig. 5

##### Etymology.

The name refers to Hebei Province, where it was collected.

##### Description.

***Sexual morph***: not observed. ***Asexual morph*: *Conidiomata*** Group AII (type a6), immersed in the bark, scattered, erumpent through the bark surface, circular, with multiple locules. ***Conceptacle*** absent. ***Disc*** conspicuous, orange to brown, circular to ovoid, 85–105 μm in diameter, with one ostiole per disc. ***Ostiole*** in the center of the disc, orange, 50–110 µm in diam. ***Locules*** numerous, subdivided frequently with independent walls, 320–820 µm. ***Conidiophores*** hyaline, branched at the base or occasionally unbranched, 12–20 × 1–1.5 (av. = 17 ± 2 × 2 ± 0.3, n = 30) µm. ***Conidiogenous cells*** enteroblastic, phialidic. ***Conidia*** hyaline, elongate-allantoid, smooth, aseptate, 3.6–6.3 × 1.0–1.5 (av. = 4.9 ± 0.4 × 1.2 ± 0.2, n = 50) μm.

##### Culture characteristics.

Cultures on PDA are initially white, growing fast up to 5 cm after 3 d and entirely covering the 6 cm Petri dish after 4 d. Colony exhibit flat elevation with moderately sparse aerial mycelium.

**Figure 5.**
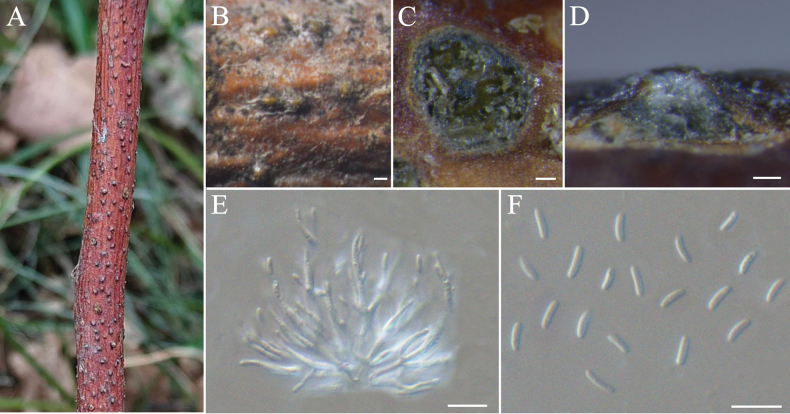
*Cytospora hebeiensis* (BJFC-S2548). **A, B.** Habit of conidiomata on branch; **C.** Transverse section through conidiomata; **D.** Longitudinal section through conidiomata; **E.** Conidiophores and conidiogenous cells; **F.** Conidia. Scale bars: 100 µm (**B–D**); 10 µm (**E, F**).

##### Materials examined.

China, Hebei Province, Saihanba, 42°23'33"N, 117°22'17"E, from branches of *Malus pumila*, 8 July 2024, C.M. Tian, T.Q. Pei & Y.Y. Wu (holotype BJFC-S2548, ex-type cultures CFCC 72601).

##### Notes.

To date, seven *Cytospora* species have been obtained from *Malus pumila* (Table 3). *Cytospora hebeiensis*, a novel species isolated from *M. pumila* in Hebei, China, can be distinguished from *C. leucostoma* by the absence of a black conceptacle (Fan et al. 2020). While the other six species also lack a black conceptacle and exhibit overlapping conidial sizes with *C. hebeiensis*, they are phylogenetically separated into distinct clades. In the phylogenetic tree (Fig. 3), *C. hebeiensis* is most closely related to *C. malvicolor* (from *Corylus mandshurica* in Beijing, China) and *C. linzhiensis* (from *Euonymus japonicus* and *Alnus nepalensis* in Xizang, China) (Lin et al. 2024; Jiang et al. 2025a). It can be distinguished from *C. malvicolor* by its smaller conidia (3.6–6.3 × 1.0–1.5 μm vs. 6.5–7.5 × 1.5–2 μm in *C. malvicolor* (Lin et al. 2024)), as well as differences in the following gene regions: ITS (3/501 bp), *act* (3/242 bp), *rpb2* (11/726 bp), *tef1-α* (21/427 bp), and *tub2* (21/426 bp). *C. hebeiensis* differs from *C. linzhiensis* at ITS (5/501 bp), *act* (4/242 bp), *rpb2* (10/721 bp), *tef1-α* (29/509 bp), and *tub2* (9/409 bp), as well as by its narrower conidia (3.6–6.3 × 1.0–1.5 μm vs. 5–6 × 1.5–2 μm in *C. linzhiensis*).

**Table 3.** Comparison of *Cytospora* species from *Malus domestica*, *M. spectabilis*, and *M. pumila*.

Species	Host	Conceptacle	Asexual (conidia)	Sexual	Reference
***Cytospora ampla***	*M. spectabilis*	absent	unknown	known	This study
* ceratosperma *	*M. pumila*	absent	4.5–5.5 × 1.0–1.5	unknown	Adams et al. 2015
*C. chrysosperma*	*M. pumila*	absent	4.0–5.6 × 0.8–1.3	known	Fan et al. 2015a
*C. cincta*	*M. domestica*	black	6.5–8.5 × 1.5–2	unknown	Norphanphoun et al. 2017
***C. hebeiensis***	*M. pumila*	absent	3.6–6.3 × 1.0–1.5	unknown	This study
*C. leucostoma*	*M. pumila*	black	4.5–5.5 × 1.0–1.5	known	Zhuang 2005
*C. mali-domesticae*	*M. domestica*	absent	4.3–7.8 × 1.2–1.7	unknown	Azizi et al. 2024
*C. mali-spectabilis*	*M. spectabilis*	absent	9.0–10.0 × 1.5–2.0	unknown	Pan et al. 2020
*C. melnikii*	*M. domestica*	absent	3.1–5 × 1.0–1.3	unknown	Azizi et al. 2024
*C. michailide siana*	*M. domestica*	absent	4.0–7.9 × 1.0–1.6	unknown	Azizi et al. 2024
*C. miyandoabensis*	*M. domestica*	absent	5.4–8 × 1.2–2	unknown	Azizi et al. 2024
*C. pistaciae*	*M. pumila*	absent	3.5–5.5 × 1.0-1–5	unknown	Daniel et al. 2020
*C. schulzeri*	*M. pumila*	absent	4.6–6.7 × 0.9–1.5	known	Fan et al. 2020
	*M. spectabilis*	absent	4.5–5.9 × 1.0–1.5	unknown	Li et al. 2024
***C. sorbariae***	*M. spectabilis*	absent	3.8–6.7 × 0.9–1.6	unknown	This study
***C. yinchuanensis***	*M. pumila*	absent	5.9–7.6 × 1.0–1.6	unknown	This study
	*M. spectabilis*			unknown	This study

#### ﻿ Cytospora pseudochrysosperma

Taxon classificationFungiDiaporthalesValsaceae

L. Lin & X.L. Fan, Studies in Mycology 109: 372 (2024)

DA8A5609-662C-518C-92A4-ABE0C4EE65F0

Fig. 6

##### Description.

***Sexual morph*: *Stromata*** Group SII, immersed in the bark, erumpent through the surface, extending to a large circular area, with 12–18 irregularly arranged perithecia. ***Conceptacle*** absent. ***Disc*** light grey to black, surrounded by ostioles, with an orange center, circular to ovoid, 123–173 µm in diam. ***Ostioles*** umber to black when mature, arranged irregularly in a disc, flask-shaped to spherical, 163–244 µm. ***Asci*** hyaline, with a chitinoid, refractive ring, clavate to elongate-obovoid, 33.5–53.2 × 7.1–10.7 (av. = 46.4 ± 1.9 × 9.4 ± 0.7, n = 30) µm, 8-spored. ***Ascospores*** hyaline, elongate-allantoid, thin-walled, aseptate, 9.3–11.8 × 1.8–2.7 (av. = 9.9 ± 0.4 × 2.2 ± 0.1, n = 50) µm. ***Asexual morph***: not observed.

##### Culture characteristics.

Cultures on PDA are white, growing fast, entirely covering the 6 cm Petri dish after 2 d, flat, retained original coloration after 30 d.

##### Materials examined.

China, Hebei Province, Saihanba, 42°23'33"N, 117°22'17"E, from branches of *Salix matsudana*, 8 July 2024, C.M. Tian, T.Q. Pei & Y.Y. Wu (BJFC-S2552, living culture CFCC 72613; BJFC-S2553, living culture CFCC 72617).

##### Notes.

*Cytospora pseudochrysosperma* was initially regarded as *C. chrysosperma* based on irregularly arranged perithecia and was confirmed to be a pathogen of poplar and willow canker disease in China (Tai 1979; Wei 1979; Zhuang 2005). Based on the phylogenetic discovery that this species resides in a clade distinct from the epitype of *C. chrysosperma*, Lin et al. (2024) established it as a new species. Jiang et al. (2025a) reported that it infects a wide range of hosts, including *Betula utilis*, *Populus* sp., and *Sorbus rehderiana*; notably, it is frequently isolated from various *Salix* spp., such as *S. takasagoalpina*, *S. caprea*, *S. integra*, and *S. chaenomeloides*. Although in the *tub2* single-gene phylogeny the sequences of *C. pseudochrysosperma* did not form a monophyletic clade (see S1), this is likely attributed to intraspecific genetic variation potentially driven by geographical differences. Crucially, in the multi-locus phylogenetic analysis, all *C. pseudochrysosperma* isolates, including strains CFCC 72613 and CFCC 72617 obtained from *S. matsudana* in this study, clustered together with a highly supported clade (MLBP/BPP = 97/1.00). This robust phylogenetic placement, coupled with congruent morphological characteristics, confirms their identification as *C. pseudochrysosperma* and extends the known host range to *S. matsudana*. This expands its previously documented locations in Gansu and Xizang to the new province, Hebei.

**Figure 6.**
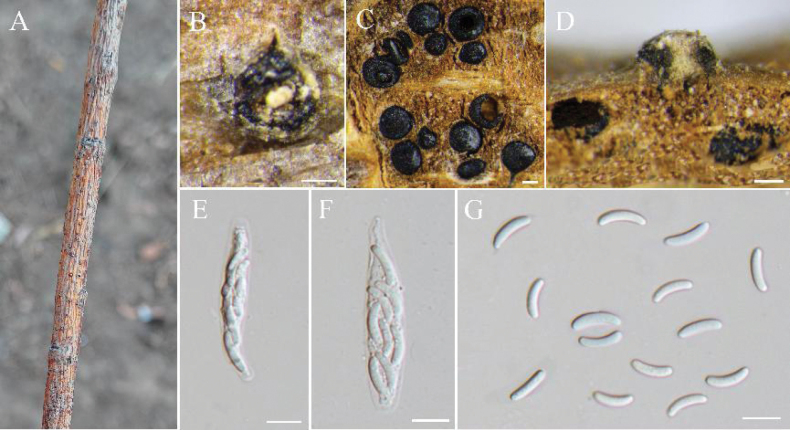
*Cytospora pseudochrysosperma* (BJFC-S2553). **A, B.** Habit of pseudostroma on branch; **C.** Transverse section through pseudostroma with ascomata; **D.** Longitudinal section through pseudostroma with ascomata; **E, F.** Asci; **G.** Ascospores. Scale bars: 100 μm (**B–D**); 10 μm (**E–G**).

#### ﻿ Cytospora sophoricola

Taxon classificationFungiDiaporthalesValsaceae

C.M. Tian & X.L. Fan, Mycoscience 55 (4): 254 (2013)

9F61644B-D1A5-5207-9C80-7CC77D24C291

Fig. 7

##### Description.

***Sexual morph***: not observed. ***Asexual morph*: *Conidiomata*** Group AIII (type a11), immersed in the bark, scattered, erumpent through the bark surface, circular to ovoid, with multiple locules. ***Conceptacle*** absent. ***Disc*** conspicuous, grey to black, circular to ovoid, 260–320 μm in diam, with one ostiole per disc. ***Ostiole*** in the center of the disc, black, 50–110 µm. ***Locules*** numerous, subdivided frequently with common walls, 106–212 µm. ***Conidiophores*** hyaline, branched at the base or occasionally unbranched, 11–16 × 0.7–1.4 (av. = 13 ± 2.1 × 1.1 ± 0.3, n = 50) μm. ***Conidiogenous cells*** enteroblastic, phialidic. ***Conidia*** hyaline, elongate-allantoid, smooth, aseptate, 5.9–7.9 × 1.5–2.0 (av. = 6.5 ± 0.4 × 1.6 ± 0.1, n = 50) μm.

##### Culture characteristics.

Cultures on PDA initially white, reaching a diameter of 5 cm after 10 d, entirely covering the 6 cm Petri dish, colonies appear flat with uneven growth, later turning tan-brown.

**Figure 7.**
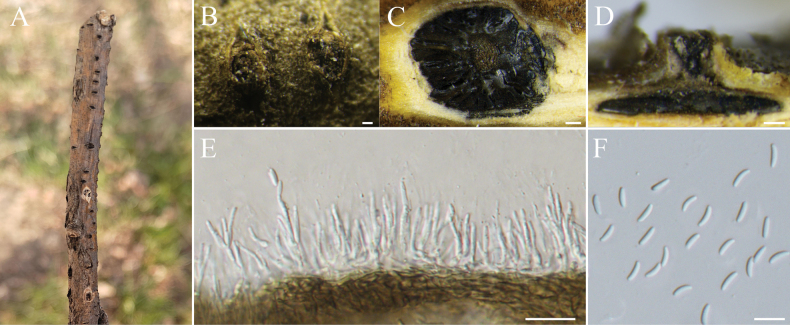
*Cytospora sophoricola* (BJFC-S2554). **A, B.** Habit of pseudostromata on branch; **C.** Transverse section through conidiomata; **D.** Longitudinal section through conidiomata; **E.** Conidiophores and conidiogenous cells; **F.** Conidia. Scale bars: 100 µm (**B–D**); 10 µm (**E, F**).

##### Materials examined.

China, Hebei Province, Saihanba, 42°23'33"N, 117°22'17"E, from branches of *Caragana microphylla*, 8 July 2024, C.M. Tian, T.Q. Pei & Y.Y. Wu (BJFC-S2554, living culture CFCC 72610).

**Notes.***Cytospora sophoricola* was first observed on branches of *Sophora japonica* in Gansu Province by Fan et al. (2014). It is similar to *C. schulzeri* (recorded from *Malus*) and *C. carbonacea* (reported from *Ulmus*), but it can be distinguished from them based on the diameters of the disc and locules, the number of ostioles, and the size of the conidia. In this study, CFCC 72610 was isolated from branches of *Caragana microphylla* and clustered in a well-supported clade (MLBP/BPP = 100/1.00) with CFCC 89595 (the ex-holotype culture of *C. sophoricola*) (Fig. 3). Therefore, CFCC 72610 is identified as *C. sophoricola*, representing a new host record for *C. sophoricola* on *Caragana microphylla* and its first documented occurrence in Hebei Province, China.

#### ﻿ Cytospora sorbariae

Taxon classificationFungiDiaporthalesValsaceae

A.L. Jia & X.L. Fan, MycoKeys 101: 184 (2024)

84392E83-F656-5DBF-8F5F-20571D9A85D0

Fig. 8

##### Description.

***Sexual morph***: not observed. ***Asexual morph*: *Conidiomata*** Group AII (type a6), immersed in the bark, scattered, erumpent through the surface of bark, with multiple locules. ***Conceptacle*** absent. ***Disc*** brown to black, circular to ovoid, erumpent through the surface of bark in a large area, conspicuous when mature, 97–241 µm in diam, with one or two ostioles per disc. ***Ostioles*** grey to black, at the same or slightly above the level of the disc surface, 60–90 µm. ***Locules*** numerous, subdivided frequently by invaginations with common walls, circular to ovoid, 160–346 µm. ***Conidiophores*** hyaline, unbranched, approximately cylindrical, 8.2–11.4 × 0.7–1.3 (av. = 9.3 ± 0.7 × 0.9 ± 0.2, n = 50) µm. ***Conidia*** hyaline, elongate-allantoid, smooth, aseptate, 3.8–6.7 × 0.9–1.6 (av. = 5.5 ± 0.7 × 1.3 ± 0.2, n = 50) µm.

##### Culture characteristics.

Cultures on PDA are initially white, with dense, flat, and radially even mycelial growth, growing fast up to cover the 6 cm Petri dish after 3 d, over time, the color of the strain darkens, turning tan.

##### Materials examined.

China, Hebei Province, Saihanba District, 42°23'33"N, 117°22'17"E, from branches of *Malus spectabilis*, 11 September 2024, C.M. Tian, T.Q. Pei & M.H. Wang (BJFC-S2555, living culture CFCC 72609).

##### Notes.

*Cytospora sorbariae*, isolated from *Sorbaria sorbifolia* in Beijing, was first introduced by Jia et al. (2024). Lin et al. (2024) extended its host range to *Corylus heterophylla* and *Betula* sp. In this study, CFCC 72609 clustered together with *C. sorbariae* with high support in the multi-gene phylogenetic tree (MLBP/BPP = 100/1.00). Therefore, we identify this isolate as *C. sorbariae*. Meanwhile, *C. sorbariae* is reported for the first time on *Malus spectabilis* and in Hebei Province, China.

**Figure 8.**
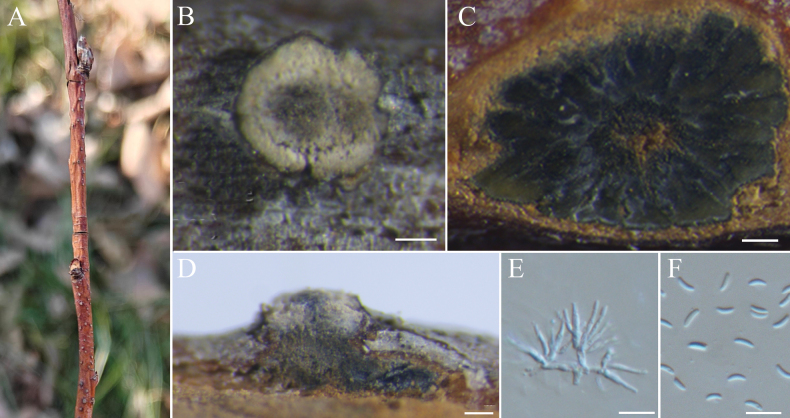
*Cytospora sorbariae* (BJFC-S2555). **A, B.** Habit of conidiomata on branch; **C.** Transverse section through conidioma; **D.** Longitudinal section through conidioma; **E.** Conidiophores and conidiogenous cells; **F.** Conidia. Scale bars: 100 μm (**B–D**); 10 μm (**E, F**).

#### ﻿ Cytospora yinchuanensis

Taxon classificationFungiDiaporthalesValsaceae

L. Lin & X.L. Fan, Studies in Mycology. 109: 393 (2024)

E028C11E-0764-5C17-B9BB-8979A8F814F6

Fig. 9

##### Description.

***Sexual morph***: not observed. ***Asexual morph*: *Conidiomata*** Group AII (type a6), immersed in bark, erumpent when mature, flat, discoid, flask-shaped to conical, with large multi-locules. ***Conceptacle*** absent. ***Disc*** light brown, circular to ovoid, 164–245 µm in diam, with one ostiole per disc. ***Ostiole*** circular to ovoid, isabelline to black, 35–45 µm. ***Locules*** subdivided frequently by invaginations with common walls, 149–299 µm. ***Conidiophores*** unbranched or branched at the bases, 13–20 × 1.5–2 (av. = 18 ± 2.3 × 1.7 ± 0.3, n = 30) µm. ***Conidia*** hyaline, unicellular, eguttulate, elongate-allantoid, 5.9–7.6 × 1.0–1.6 (av. = 6.3 ± 0.2 × 1.3 ± 0.1, n = 50) µm.

##### Culture characteristics.

Cultures initially white, covering the entire 6 cm Petri dish within 2 d, exhibiting radially uniform growth; no color change observed at 4 d, with mycelium becoming denser after 7 d.

##### Materials examined.

China, Hebei Province, Saihanba District, 42°23'33"N, 117°22'17"E, from branches of *Malus spectabilis*, 11 September 2024, C.M. Tian, T.Q. Pei & M.H. Wang (BJFC-S2556, living culture CFCC 72611); 42°23'33"N, 117°22'17"E, from branches of *M. pumila*, 11 September 2024, C.M. Tian, T.Q. Pei & M.H. Wang (BJFC-S2557, living culture CFCC 72612).

##### Notes.

*Cytospora yinchuanensis* was initially misidentified as *C. schulzeri* based on its numerous ostioles and erumpent pycnidia (Fan et al. 2020) and was reported to cause canker and dieback disease. Lin et al. (2024) isolated this strain from *Malus pumila* in Ningxia and first described it as a new species, *C. yinchuanensis*, based on phylogenetic inference. In this study, two isolates (CFCC 72611 and CFCC 72612) grouped together with *C. yinchuanensis* (MLBP/BPP = 100/1.00), and their morphological characteristics are similar. Therefore, they are identified as *C. yinchuanensis*, representing a new host record on *M. spectabilis* and the first record in Hebei Province.

**Figure 9.**
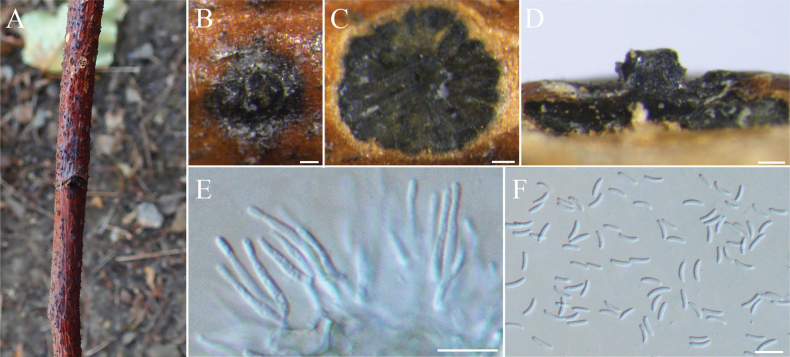
*Cytospora yinchuanensis* (BJFC-S2557). **A, B.** Habit of conidiomata on branch; **C.** Transverse section through conidioma; **D.** Longitudinal section through conidioma; **E.** Conidiophores and conidiogenous cells; **F.** Conidia. Scale bars: 100 μm (**B–D**); 10 μm (**E, F**).

## ﻿Discussion

This study describes and illustrates six species of *Cytospora* from Hebei Province, China, for the first time. These species reside in two species complexes. The *Chrysosperma* SC comprises a new host record, and the *Ribis* SC comprises four new host records and one new species, namely *C. hebeiensis*.

*Cytospora* was introduced with *C. chrysosperma* as the type species (Ehrenberg 1818). Infected branches or twigs are characterized by elongate, slightly sunken, and discolored areas and often split along the canker margin (Fan et al. 2020). Meanwhile, the morphs of *Cytospora* are identified by single or labyrinthine locules (and diaporthalean-like perithecia), filamentous conidiophores (and clavate to elongate obovoid asci), and allantoid, hyaline conidia (and ascospores) (Spielman 1983, 1985; Adams et al. 2005).

Early taxonomic studies of ascomycetes primarily relied on ITS single-gene phylogenetics. However, this approach often failed to distinguish between closely related species within several genera, such as *Colletotrichum* and *Diaporthe* (Weir et al. 2012; Gomes et al. 2013); thus, a multi-locus phylogenetic method was introduced to resolve this issue. In recent years, it has been widely applied to the identification of ascomycetes, and many scholars have identified pathogens on different hosts such as *Rosa* and *Larix* by combining ITS and other gene regions, which has further enriched the understanding of the diversity of fungi associated with various plants (Vu et al. 2019; Peng et al. 2022; Xiao et al. 2023; Dewing et al. 2025; Jiang et al. 2025b, c; Zhu et al. 2025). Given that *Cytospora* species are quite similar to each other in morphology, this method is applicable for reliable identification. Accordingly, many researchers have successfully employed a five-gene system (ITS, *act*, *rpb2*, *tef1-α*, and *tub2*) for phylogenetic analyses to achieve accurate *Cytospora* species discrimination.

The phenomenon of a single host being infected by multiple congeneric fungal species is ubiquitous. Norphanphoun et al. (2025) reported a complex of three *Colletotrichum* species (*C. actinidicola*, *C. poalesicola*, and *C. karsti*) on *Rosa chinensis*. In the walnut pathosystem isolated seven *Diaporthe* species associated with stem blight and demonstrated via pathogenicity assays that all were pathogenic, causing blight and dieback. Likewise, Azizi et al. (2024) isolated five *Cytospora* species from cankered apple stems and branches. These studies collectively demonstrate that a single host can be infected by more than one species. In the present study, we summarized a total of 15 reported *Cytospora* species on *Malus* spp (Table 3), including a newly identified species in this study. This finding significantly contributes to the understanding of *Cytospora* diversity associated with apples in China.

The discovery and description of novel species is a fundamental component of advancing our knowledge of fungal diversity (Ariyawansa et al. 2020; Crous et al. 2023a, b). Although studies on *Cytospora* have increased in recent years (Fan et al. 2014; Jiang et al. 2020; Jia et al. 2023; Azizi et al. 2024; Lin et al. 2024; Jiang et al. 2025a), relatively few fresh specimens have been obtained from Hebei. Up to now, only a limited number of species, such as *C. microspora* and *C. personata*, have been documented (Fan et al. 2020). The Saihanba Region in Hebei is characterized by monoculture plantations with a rich shrub understory, a combination that increases its vulnerability to economically significant disease outbreaks. While the findings of this study provide crucial data for formulating effective control measures and substantially enhance our knowledge of *Cytospora* diversity in this unique ecosystem, more extensive sampling remains necessary to better delineate the *Cytospora* taxa in this region.

## Supplementary Material

XML Treatment for Cytospora ampla

XML Treatment for Cytospora hebeiensis

XML Treatment for Cytospora pseudochrysosperma

XML Treatment for Cytospora sophoricola

XML Treatment for Cytospora sorbariae

XML Treatment for Cytospora yinchuanensis

## ﻿Additional information

### Use of AI

No use of AI was reported.

### Author ORCIDs

Tingqian Pei https://orcid.org/0009-0004-0073-7608

Dianguang Xiong https://orcid.org/0000-0001-5737-1476

Yingmei Liang https://orcid.org/0000-0002-1690-5512

### Data availability

All of the data that support the findings of this study are available in the main text or Supplementary Information.

## Supplementary materials

Supplementary material 1 Single-locus phylogenetic tree of the *Chrysosperma* SC and *Ribis* SC Data type rar Explanation note **figs S1–S5.** Single-locus phylogenetic tree of the *Chrysosperma* SC; **figs S6–S10.** Single-locus phylogenetic tree of the *Ribis* SC. https://binary.pensoft.net/file/1496491This dataset is made available under the Open Database License (http://opendatacommons.org/licenses/odbl/1.0/). The Open Database License (ODbL) is a license agreement intended to allow users to freely share, modify, and use this Dataset while maintaining this same freedom for others, provided that the original source and author(s) are credited.Tingqian Pei, Dianguang Xiong , Yingmei Liang

Supplementary material 2 Strains of *Cytospora* used in the molecular analyses in the *Chrysosperma* SC Data type xlsx Explanation note Strains of species isolated from this study are shown in black bold, NA: notapplicable. Strains in this study are marked in bold, and * indicates ex-type strains. https://binary.pensoft.net/file/1496492This dataset is made available under the Open Database License (http://opendatacommons.org/licenses/odbl/1.0/). The Open Database License (ODbL) is a license agreement intended to allow users to freely share, modify, and use this Dataset while maintaining this same freedom for others, provided that the original source and author(s) are credited.Tingqian Pei, Dianguang Xiong , Yingmei Liang

Supplementary material 3 Strains of *Cytospora* used in the molecular analyses in the *Ribis* SC Data type xlsx Explanation note Strains of species isolated from this study are shown in black bold, NA: notapplicable. Strains in this study are marked in bold, and * indicates ex-type strains. https://binary.pensoft.net/file/1496493This dataset is made available under the Open Database License (http://opendatacommons.org/licenses/odbl/1.0/). The Open Database License (ODbL) is a license agreement intended to allow users to freely share, modify, and use this Dataset while maintaining this same freedom for others, provided that the original source and author(s) are credited.Tingqian Pei, Dianguang Xiong , Yingmei Liang
